# Impact of level and patterns of alcohol drinking on coronary heart disease and stroke burden in Argentina

**DOI:** 10.1371/journal.pone.0173704

**Published:** 2017-03-10

**Authors:** Ariel Esteban Bardach, Joaquín Enzo Caporale, Adolfo Luis Rubinstein, Goodarz Danaei

**Affiliations:** 1 Institute for Clinical Effectiveness and Health Policy’, Buenos Aires, Argentina; 2 National Scientific and Technical Research Council (CONICET)–Buenos Aires, Argentina; 3 Department of Global Health and Population–Department of Epidemiology, Harvard School of Public Health, Boston, United States of America; University of Bologna, ITALY

## Abstract

**Background:**

Deaths from cardiovascular disease (CVD), including coronary heart disease (CHD) and stroke are expected to increase in Latin America. Moderate and regular alcohol consumption confers cardiovascular protection, while binge drinking increases risk. We estimated the effects of alcohol use on the number of annual CHD and stroke deaths and disability-adjusted life years (DALYs) in Argentina.

**Methods:**

Alcohol use data were obtained from a nationally representative survey (EnPreCosp 2011), and etiological effect sizes from meta-analyses of epidemiological studies. Cause-specific mortality rates were from the vital registration system.

**Results:**

There were 291,475 deaths in 2010 including 24,893 deaths from CHD and 15,717 from stroke. 62.7% of men and 38.7% of women reported drinking alcohol in the past year. All heavy drinkers (i.e. women who drank >20g/day and men who drank >40g/day of alcohol) met the definition of binge drinking and therefore did not benefit from cardioprotective effects. Alcohol drinking prevented 1,424 CHD deaths per year but caused 935 deaths from stroke (121 ischemic and 814 hemorrhagic), leading to 448 CVD deaths prevented (58.3% in men). Alcohol use was estimated to save 85,772 DALYs from CHD, but was responsible for 52,171 lost from stroke.

**Conclusions:**

In Argentina, the cardioprotective effect of regular and moderate alcohol drinking is slightly larger than the harmful impact of binge drinking on CVD. However, considering global deleterious effects of alcohol in public health, policies to reduce binge drinking should be enforced, especially for young people. Studies are still needed to elucidate effects on cardiovascular health.

## Introduction

Worldwide, non-communicable diseases caused 36 million deaths in 2010, accounting for 63% of all deaths. Cardiovascular disease (CVD) deaths are increasing globally [1] and cause almost 17 million deaths each year, the majority of which occur in low and middle-income countries.[2] In Latin America, coronary heart disease (CHD) and stroke cause 42.5% and 28.8% of the CVD mortality, respectively [3] and it is estimated that between 1990 and 2020, deaths from CVD, including coronary heart disease, will increase by approximately 145%, compared with an increase of 28% for women and 50% for men in developed countries during the same period.[4] Between half to three quarters of the risk can be explained by risk factors such as an unhealthy diet, low physical activity and tobacco use.[5] In Argentina, 97,107deaths (30.5% of all deaths) were due to CVD in 2010[6] and CHD and stroke are estimated to cause 600,000 DALYs lost each year. [7]

Alcohol use was one of the most important risk factors for the overall disease burden in 2010 in Latin America.[5] However, epidemiological studies have been scarce in most of its countries.[8] Alcohol consumption has been a deeply ingrained habit across the region. The region of Southern Latin America (Chile, Argentina and Uruguay), registers an estimated per-adult alcohol consumption higher than other regions of Latin America, Asia, Africa and Oceania, but lower than Europe and similar to North America.[9] Consumption has remained stable at a relatively high level since 1990.[10] For low-income countries, a strong relation between economic wealth and alcohol consumption has been shown: the higher the gross domestic product, the higher the overall volume of consumption and the lower the proportions of abstainers. [11]

In some countries of the Latin America alcohol consumption is the leading risk factor in terms of DALYs, causing over 10.0% of the DALYs in the region. Moreover, it is increasing for females, going from fifth place in 1990 to fourth in 2010 for the Latin America and Caribbean region. [12,13]

Alcohol is a contributing cause for over 200 diseases. The relation is mostly a dose-response type for most conditions; the more alcohol consumed, the greater the disease risk. However, regular light alcohol consumption seems to confer protective effects on coronary heart disease and ischemic stroke as evidenced in several large meta-analyses of observational studies. [14] This protective effect disappears in individuals with binge drinking.[15] Binge drinking also increases the risk of hemorrhagic stroke. [16]

Burden of disease estimates for alcohol use and related conditions, such as CVD, have not been previously described in detail for adults for Argentina. We therefore aimed to estimate the effects of alcohol use on annual CHD and stroke deaths and disability-adjusted life years (DALYs) using a recent nationally representative survey.

## Methods

We conducted a comparative risk assessment analysis applied to the Argentinean population to estimate deaths from CHD and stroke that would be prevented in 2010 for subjects aged 35 and older, using as the comparator a hypothetical scenario where nobody would drink alcohol. The main inputs to the analysis were the current prevalence of alcohol use according to different consumption patterns, the etiological effect of alcohol use on disease-specific mortality, the case-fatality rates from specific diseases and the total number of disease-specific deaths in the population.

### Data sources

We used the EnPreCosp 2011 to measure the quantity of alcohol consumed and the pattern of consumption. EnPreCosp is a multi-stage, stratified, cluster-sampling, nationally representative survey of 34,343 non-institutionalized Argentinean adults aged 16 to 65 years. [17] We obtained detailed information on frequency, amount, and type of alcoholic beverage consumed. Average daily alcohol consumption was estimated using a questionnaire focused on both the usual quantity and frequency of drinking, as well as quantity and frequency of binge drinking. We accounted for complex survey design using sampling weights.

Categorical levels for average volume of pure alcohol per day were selected to be consistent with previous meta-analyses[18–20] and were defined as follows: abstainer as a person who does not report having had a drink containing alcohol within the last year; regular drinker as women who drink less than 20g/day of alcohol and men drinking less than 40 g/day who don’t engage in binge drinking; and binge drinking was defined as having at least one occasion of five or more drinks in the last month. All subjects falling above these levels met the definition of binge drinkers in the survey and were thus categorized as so. We imputed level of alcohol use in those aged 66 years and more using a multinomial logistic regression.

The relative risk (RR) for each exposure category for CHD[21–23], ischemic stroke and hemorrhagic stroke[21,23–25] were derived from high-quality published systematic reviews and meta-analyses of epidemiological studies (Table 1).

**Table 1:** Relative risks of the effects of alcohol use on disease-specific mortality.

Diseases Outcome	Sex	Age groups	Regular Drinkers	Binge Drinkers		Source of RR
Coronary Heart Disease	M	35 to 44	0.6	1		Refs 15–16, 21–23
	F	35 to 44	0.6	1		
	M	45 to 59	0.63	1		
	F	45 to 59	0.63	1		
	M	60 to 69	0.82	1		
	F	60 to 69	0.82	1		
	M	70 to 79	0.92	1		
	F	70 to 79	0.92	1		
	M	80+	0.97	1		
	F	80+	0.97	1		
Ischemic Stroke	M	35 to 44	0.83	1.41		Refs 21,23–24
	F	35 to 44	0.88	1.41		
	M	45 to 59	0.83	1.41		
	F	45 to 59	0.88	1.41		
	M	60 to 69	0.94	1.41		
	F	60 to 69	0.96	1.41		
	M	70 to 79	0.97	1.41		
	F	70 to 79	0.98	1.41		
	M	80+	1	1.41		
	F	80+	1	1.41		
Hemorrhagic Stroke	M	35 to 44	1.65	2.01		Refs 23–25
	F	35 to 44	1.3	2.01		
	M	45 to 59	1.65	2.01		
	F	45 to 59	1.3	2.01		
	M	60 to 69	1.19	2.01		
	F	60 to 69	1.09	2.01		
	M	70 to 79	1.09	2.01		
	F	70 to 79	1.04	2.01		
	M	80+	1	2.01		
	F	80+	1	2.01		

Vital registration data from the year 2010 was used to derive the annual cause-specific mortality. [26] We used ICD10 codes: I20-I25 for CHD, I60.x-I62.x for hemorrhagic stroke and I63.x for ischemic stroke. Since the majority of stroke deaths in death certificates in the country do not differentiate stroke by ischemic or hemorrhagic type, we allocated codes I64.x/I67.x/I69.x proportionally to each stroke type according to the proportion of subtypes observed in hospital-based stroke registries described by Saposnik et al. [27,28]

We reallocated deaths that were assigned to poorly-defined (garbage) codes. These included (1) 24,484 deaths that were assigned to "symptoms, signs and poorly defined conditions” (R00-R99); and (2) 29,439 deaths that were assigned to ill-defined CVD codes (I47.2, I49.0, I46, I50, I51.4, I51-5, I51.6, I51.9, I70.9). We reallocated 21.5% of garbage codes to ischemic heart disease as estimated in the Global Burden of Disease (GBD) 2010 project. [29]

### Estimating event rates

Considering lack of local data, in order to estimate age and gender-specific fatal- and-nonfatal event rates derived from CVD deaths we divided cause-specific mortality rates (by age and gender) by case fatality rates. This approximation has been widely used in epidemiologic and health economic models by the World Health Organization in tools such as DisModII or the WHO-CHOICE and GLOBOCAN[30,31] among others. In the case of CHD, total fatal and non-fatal acute myocardial infarctions (AMI) and acute coronary syndromes (ACS) were estimated from the official national number of CHD deaths for 2010[26] and 28-day case-fatality rate for AMI for Southern Latin America (38% in women; 44% in men) [29]; for ACS we considered case-fatality rates as one-third of those of AMI, according to local sources. [32] For stroke, total events (hemorrhagic and ischemic ones, fatal and non-fatal) were estimated in the same manner from national data on stroke deaths and from case fatality rates obtained from the national hospital discharge registry for the public sector. [33] We assumed that the age distribution of events presented in the public hospitals discharge registry was representative of that of Argentina as a whole (including the private sector). S1C and S1D Table show these estimations.

### Estimating mortality attributable to alcohol

For CHD and stroke separately by subtype, we computed the proportional changes in disease-specific deaths that would occur if nobody in the population drank alcohol, using the formula below[34]

$$PAF=\frac{\int_{x=0}^{m}RR(x)P(x)dx-\int_{x=0}^{m}RR(x)P^{\prime }(x)dx}{\int_{x=0}^{m}RR(x)P(x)dx}$$

Where PAF is the Population Attributable Fraction, x = exposure level; P(x) = actual distribution of exposure in the population; P’(x) = alternative distribution of exposure in the population; RR(x) = relative risk of cause-specific mortality at exposure level x; and m = maximum exposure level.

As alcohol use was a risk measured in categories, we used the discrete version of the same estimator.

### Calculation of disability-adjusted life years (DALY)

We estimated undiscounted DALYs for acute myocardial infarction (AMI) (I21, I22), acute coronary syndrome (ACS) (I20, I24, I25) and stroke (I60-I64, I67, I69). DALYs lost to hemorrhagic and ischemic stroke were calculated according to the distribution of deaths from these causes. We followed the GBD Study method[35,36] considering Fox-Rushby individual equations[37] for Years of Life Lost (YLL) and Years of Life with Disability (YLD). YLLs were calculated taking into account life expectancy in Argentina. [38] Duration for YLDs were estimated using DISMOD II[39], it was assumed to be the same for AMI and ACS. All disability weights (DW) were obtained from Salomon et al 2012. [36] We calculated a unique weight for each of the conditions included (AMI, ACS and Stroke). For AMI we weighted DWs from days 1–2 and days 3–28 according to the distribution of lenght of stay of hospitalizations due to AMI in the public sector. [40] For ACS we weighted the DWs using data on proportion of cases at different severity levles from a large Spanish coronary heart disease registry. [41] In the case of stroke we multiplied the DW of each category of severity by the corresponding distribution of cases reported in Argentinean literature from hospital data. [42] We mapped each DW to their corresponding category of the modified Rankin Scale (mRS) assuming similar distribution of severity on the basis of the GBD sequel definition. The mRS is a commonly used scale for measuring the degree of disability or dependence in the daily activities of people who have suffered a stroke or other causes of neurological disability. The slight difference between the definition of disability on mRS and categories in GBD 2010 was regarded as acceptable by a consensus of members of an international collaborative stroke expert group from a study performed in South Africa, that used the same methodology. [43] Details of DWs calculations are shown in the S1F Table.

## Results

**Fig 1:**
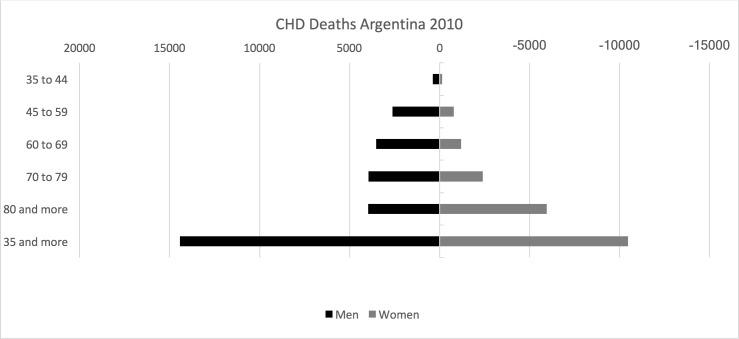
CHD and Stroke Deaths by age groups, Argentina 2010.

There were 291,475 deaths in 2010. CHD accounted for 24,893 deaths and stroke for another 15,717 (Fig 1). 62.7% of men and 38.7% of women reported drinking alcohol in the past year. All women who drink more than 20g/day of alcohol and men drinking more than 40 g/day met the definition of binge drinkers (Table 2). Binge drinking occurred mostly during weekends. In addition, binge drinking was more common among beer drinkers (18.7% of men, and 13.8% in women) compared with wine drinkers (12.3% in men and 9.1% in women) or spirits drinkers (2.3% and 2.0%, respectively). Two-thirds of binge drinkers were younger than 35 years old both in men and women (Table 2) and 38% had education level less than high-school (38.2% of people in that category).

**Table 2:** Population exposures to alcohol (prevalence percentages). Argentina. EnPreCosp survey 2011.

	Abstainers	Regular	Binge
		Drinkers	
Men		
35 to 49	35.2	40.1	24.8
50 to 64	37.0	45.2	17.8
65 to 79	37.9	49.6	12.5
80+	38.0	54.1	7.8
35+	37.0	47.3	15.7
Women		
35 to 49	61.2	34.7	4.1
50 to 64	65.7	32.2	2.0
65 to 79	69.3	29.7	1.0
80+	73.2	26.3	0.4
35+	67.4	30.7	1.9

Abstainer, a person not having had a drink containing alcohol within the last year.

Regular drinker, 0–<20 g of pure alcohol daily (females) and 0–<40 g (males) and not having five or more drinks in any occasion in the last month.

Binge drinking: having at least one occasion of five or more drinks in the last month.

**Fig 2:**
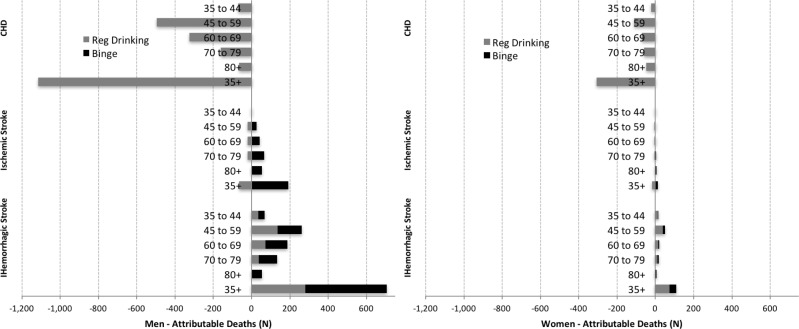
CVD deaths attributable to alcohol consumption by gender, Argentina 2010.

Alcohol use prevented 1,424 CHD deaths in adults of 35 years and older, but also caused 935 deaths from stroke (121 ischemic and 814 hemorrhagic) (Fig 2). The mortality burden of alcohol occurred due to higher stroke deaths mainly among binge-drinkers: the total number of attributable CVD deaths due to binge-drinking was 663 (92.6% occurring in men). In contrast, in regular drinkers, the protective effects on CHD mortality were larger than the harmful effects on stroke, leading to a net benefit or overall saving of 488 deaths. 58% of CHD and stroke deaths attributable to alcohol use occurred in men, in particular due to larger number of attributable deaths from hemorrhagic stroke (87% of total stroke deaths) as men consumed more alcohol and had more binge drinking (Table 3).

**Table 3:** Population attributable fractions (PAF) for ischemic heart disease, ischemic stroke and hemorrhagic stroke, and number of corresponding alcohol-attributable deaths by gender according to level and pattern of drinking. Argentina, 2010.

		Men					Women					Both genders		
	Age range	Regular drinkers		Binge		Total	Regular drinkers		Binge		Total	Regular drinkers	Binge	Total
		PAF(%)	Attributable	PAF(%)	Attributable	Attributable	PAF(%)	Attributable	PAF(%)	Attributable	Attributable	Attributable	Attributable	Attributable
			Deaths (n)		Deaths (n)	Deaths (n)		Deaths (n)		Deaths (n)	Deaths (n)	Deaths (n)	Deaths (n)	Deaths (n)
Coronary Heart Disease	35 to 44	-19	-70	0	0	-70	-16	-21.9	0	0	-21.9	-91.9	0	-91.9
	45 to 59	-19	-494.9	0	0	-494.9	-14	-109.8	0	0	-109.8	-604.7	0	-604.7
	60 to 69	-9	-323.9	0	0	-323.9	-6	-70.2	0	0	-70.2	-394.1	0	-394.1
	70 to 79	-4	-161.5	0	0	-161.5	-2	-58.8	0	0	-58.8	-220.3	0	-220.3
	80+	-2	-65	0	0	-65	-1	-47.5	0	0	-47.5	-112.5	0	-112.5
	Total	-8	-1115.3	0	0	-1115.3	-3	-308.2	0	0	-308.2	-1423.5	0	-1423.5
Ischemic Stroke	35 to 44	-7	-2.3	9	2.9	0.6	-4	-1	2	0.4	-0.6	-3.3	3.3	0
	45 to 59	-6	-21.2	8	25.8	4.6	-3	-5.6	1	1.6	-4	-26.8	27.4	0.6
	60 to 69	-3	-21.3	6	41.5	20.2	-1	-5	1	2.1	-2.9	-26.3	43.6	17.3
	70 to 79	-2	-20.5	5	66.6	46.1	-1	-6.7	<1	4.5	-2.2	-27.2	71.1	43.9
	80+	0	0	3	54.2	54.2	0	0	<1	5.2	5.2	0	59.4	59.4
	Total	-3	-65.3	5	191	125.7	-1	-18.3	2	13.8	-4.5	-83.6	204.8	121.2
Hemorrhagic Stroke	35 to 44	21	35.3	20	33.3	68.6	9	12.2	3	4.3	16.5	47.5	37.6	85.1
	45 to 59	18	135.8	17	126.6	262.4	7	37.9	2	11.8	49.7	173.7	138.4	312.1
	60 to 69	8	73.5	13	114.4	187.9	3	14.2	1	6.4	20.6	87.7	120.8	208.5
	70 to 79	4	36.9	11	95.9	132.8	1	9.6	1	7.5	17.1	46.5	103.4	149.9
	80+	0	0	7	53.2	53.2	0	0	<1	5.1	5.1	0	58.3	58.3
	Total	10	281.5	12	423.4	704.9	4	73.9	1	35.1	109	355.4	458.5	813.9

As regards the health impact according to level of education, the greatest savings in the number of attributable deaths in coronary heart disease were seen in people with complete primary and incomplete high school, in both genders. Accordingly, the highest number of attributable deaths for both ischemic and hemorrhagic strokes were observed within the same category. The second group in importance, both for health gains and losses was the one with the highest educational level (see S1G Table for details).

**Fig 3:**
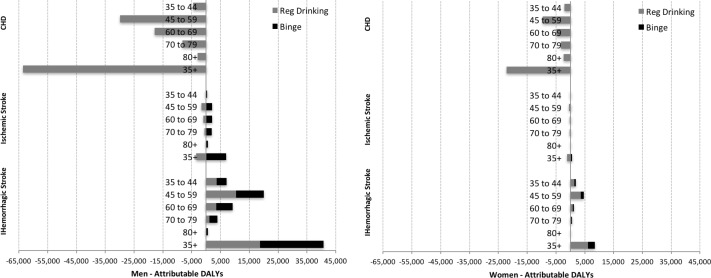
CVD DALYs attributable to alcohol consumption by gender, Argentina 2010.

Alcohol use saved 85,772 DALYs from CHD, more in men (74.2%) than in women but was responsible for 52,171 DALYs from stroke (84.8% in men). Overall 33,551 CVD DALYS were averted (Fig 3). The ‘premature death’ (i.e. YLL) component of CHD in men explained this averted total burden (see S1B Table). Regular drinking explained the averted burden mainly by CHD condition and mostly in men (Table 4).

**Table 4:** DALYs lost/averted to alcohol intake by level and pattern of drinking, age and gender ^1^^.^

CVD Condition	Age range	Men			Women			Both genders		
		Regular	Binge	Total	Regular	Binge	Total	Regular	Binge	Total
		Drinkers			Drinkers			Drinkers		
Coronary Heart Disease	35 to 44	-4,554	0	-4,554	-2,019	0	-2,019	-6,573	0	-6,573
	45 to 59	-29,940	0	-29,940	-9,648	0	-9,648	-39,588	0	-39,588
	60 to 69	-17,864	0	-17,864	-4,859	0	-4,859	-22,723	0	-22,723
	70 to 79	-8,236	0	-8,236	-3,238	0	-3,238	-11,474	0	-11,474
	80+	-3,006	0	-3,006	-2,358	0	-2,358	-5,364	0	-5,364
	Total	-63,600	0	-63,600	-22,122	0	-22,122	-85,722	0	-85,722
Ischemic Stroke	35 to 44	-235	300	65	-119	42	-77	-354	342	-12
	45 to 59	-1,633	2,002	369	-519	153	-366	-2,152	2,155	3
	60 to 69	-1,027	2,053	1,026	-279	116	-163	-1,306	2,169	863
	70 to 79	-603	1,969	1366	-238	159	-79	-841	2,128	1,287
	80+	0	633	633	0	76	76	0	709	709
	Total	-3,498	6,957	3,459	-1,155	546	-609	-4,653	7,503	2,850
Hemorrhagic Stroke	35 to 44	3,623	3,418	7,041	1,420	499	500,42	5,043	3,917	8,960
	45 to 59	10,388	9,695	20,083	3,569	1,156	4,725	13,957	10,851	24,808
	60 to 69	3,56	5,594	9,154	833	370	1203	4,393	5,964	10,357
	70 to 79	1,082	2,819	3,901	347	261	608	1,429	3,080	4,509
	80+	0	623	623	0	64	64	0	687	687
	Total	18,653	22,149	40,802	6,169	2,350	8,519	24,822	24,499	49,321

^1^ A negative figure indicates DALYs averted while positive are DALYs lost. Regular drinking averts DALYs for CHD and IS at both gender and for all age groups; these DALYs averted compensate DALYs lost due to HS. Both DALYs, averted or lost, generally have a maximum at the 45 to 59 age group and decreased across older age groups.

## Discussion

We estimated that alcohol use averted 1424 deaths from CHD, representing a reduction of 6% in CHD deaths, but caused 121 ischemic stroke deaths and 814 deaths from hemorrhagic stroke in 2010 in Argentina, which amount to 6% of total stroke deaths. About half of the adult population reported drinking alcohol in the last month, and roughly half of them reported binge drinking, mostly on weekends. We estimated that alcohol use saved 85,772 DALYs from CHD but caused 52,171 DALYs due to stroke. 58% of CVD deaths attributable to alcohol occurred in men, particularly among binge drinkers.

Although much dependent on the levels and patterns of drinking, and with variations across time and countries, the imbalanced risk in terms of gender in cardiovascular and other mortality burden attributable to alcohol observed in our study has been established before. [44–46] The GBD project estimates that alcohol use was responsible of 0.75% of total DALYs for Argentina in 2013. [10] However, we were not able to compare our results directly, as the country-specific and cause-specific risk factor estimates were not publicly available.[12] It was previously estimated for the region of the Americas that about 5% of attributable deaths were due to alcohol.[11] In Brazil, for example, 13% of DALYs in men and 3% in women were attributed to alcohol in 2004.[11] The only previous Argentinean study on disease burden[47] estimated that for 2005 the total number of YLLs from all causes attributed to alcohol was 7,263 in men and 641 in women, with a peak between 45 and 64 years of age, for both genders. The number of total DALYs from this causes was 49,358 in men and 29,242 in women, totaling 78,600 DALYs, which represented 2.63% of the total burden of disease.

Our study has a number of strengths: effect sizes were derived from large meta-analyses of observational studies that had adjusted for important confounders. We presented attributable deaths by age, sex, and by drinking pattern. We used recent national data on mortality with garbage code redistribution and local data for the estimation of case fatality ratios. Also, the estimated CFRs were in line with those reported in a published large meta-analysis of population-based studies for low- and middle-income countries for early (21 days to 1 month) stroke CFR. [48]

The main limitation of our work is related to its focus on CVD: we did not consider other health consequences of alcohol intake such as its protective effect on diabetes or its harmful impact on violence, injuries, cancers and gastrointestinal and psychiatric diseases, scope for a future publication. Alcohol abuse disorder has been estimated in other Latin American countries as one of the major causes of disease burden: in Peru, one study found it to be the third cause of total DALYs in 2004;[49] and in Chile, another study projected it to become the fifth cause of lost DALYs by 2020.[50] Another limitation of our study is that RRs from high-income countries may not be generalizable to middle-income countries such as Argentina. Nevertheless, such extrapolation is required to inform policy-making in the absence of local prospective studies with sufficient follow-up to derive effect sizes. Separating the relative risks by drinking pattern (i.e. binge versus regular) makes this extrapolation more justifiable as a major reason for differences in relative risks across populations is different patterns of drinking. The mortality risk of lifetime abstainers do appear to be slightly lower than that of current abstainers[51]; however, the proportion of former drinkers among abstainers was small in this survey (5–10% in different age categories), not altering our estimates in a significant way. The survey used in our analysis may have underestimated alcohol use as the questionnaire did not include a direct reference to intake of Fernet, an herbal alcoholic drink that is commonly consumed especially in some provinces, although less than one percent of people consuming alcohol in EnPreCosp referred consuming “other alcoholic drinks [not beer, wine or spirits]”.

Public policies may reduce alcohol use and improve drinking patterns, and thus have a substantial preventive capacity. Several policies have been implemented in other countries to prevent the harm caused by alcohol such as imposing extra taxes on alcoholic drinks or imposing minimum sales prices, reducing availability, reducing hours of sale, and measures to limit drinking and driving. [52,53] Also, the regulation of alcohol advertisements may play an important role. [54] These may help reducing the harmful use of alcohol.

Alcohol marketing regulations in the Americas are relatively weak. Currently, most countries have no statutory regulations for marketing alcohol on national television, radio or the internet. Marketing strategies include restriction of promotion of alcohol for young people and women; packaging measures; life-style marketing and sponsorships and use of the social media. [55]

In Argentina, several policies to reduce alcohol intake have been implemented in the last years, including enforcing compliance with an upper limit of blood alcohol concentration for drivers through random alcohol checks in several large cities. Countrywide, alcoholic beverages are taxed between 8 to 20%, with the exception of wine which is not taxed, contrary to what happens in other countries with a wine industry. A national prevention program that has been in place since 2010 aims to reduce heavy and binge drinking by promoting educational activities, actions directed at health services like early detection of conditions linked to excessive consumption of alcohol, training health professionals to identify drinkers at risk. The law also mandates the Social Security System to cover all costs of medical, pharmacological and psychological services related to treatments of alcohol-related disorders. The law is of national enforcement, and the provinces adhering determine the competent local authorities to apply sanctions for noncompliance. Of note, this law does not necessarily reflect the best evidence prevention practices, and compliance with it may still be quite low. EnPreCosp 2011 showed that some wine-industrial provinces like Mendoza, San Juan but also Buenos Aires, bear the highest per-capita consumption.

Partly due to the above policies, alcohol consumption per capita has been steadily declining in Argentina, reaching less than 10 liters of pure alcohol per capita per year in 2010 compared with almost 20 liters in 1970. [54] However, our results indicate that there is still an urgent need to reduce the consumption of alcohol especially binge drinking among younger individuals. The National Survey of Risk Factors in 2013 showed that heavy regular consumption (>40g per day in men and >20 gr per day in women) decreased slightly (from 10.7% to 9.7%) as did the percentage of people driving under the effects of alcohol (from 13.2% to 12.1%) while binge drinking rose (from 8.9% to 10.4%) compared with 2009. [56]

### Conclusion

In Argentina, the beneficial effects of regular alcohol use on CHD are only slightly larger than the harmful impact of binge drinking and on stroke. Policies and programs to reduce binge drinking should be enforced at national and province levels.

## Supporting information

S1 Table a. Distribution of cause-specific deaths attributable to alcohol by age group and by sex. b. Years of life lost (YLL) and years of life with disability (YLD) by age group, gender and health condition. c. Incident CVD cases estimated (fatal and non-fatal). d. Case-fatality rates used for estimating CHD and stroke incident cases. e. Stroke disability distribution from an Argentinean source (Aleman et al, 2014). f. Stroke disability categories and corresponding DWs. g. Number of alcohol-attributable deaths for ischemic heart disease, ischemic stroke and hemorrhagic stroke, by educaction level and gender. Argentina, 2010.
(DOCX)

## References

[1] World Health Organization. The global burden of disease: 2004 update. Geneva. 2008. Available at: http://www.who.int/healthinfo/global_burden_disease/GBD_report_2004update_full.pdf. Accessed April 30th, 2015.

[2] Gersh BJ, Sliwa K, Mayosi BM, Yusuf S. Novel therapeutic concepts: the epidemic of cardiovascular disease in the developing world: global implications. European heart journal 2010; 31(6): 642–8. 10.1093/eurheartj/ehq030 20176800

[3] Fernando L, Pamela S, Alejandra L. Cardiovascular disease in Latin America: the growing epidemic. Progress in cardiovascular diseases 2014; 57(3): 262–7. 10.1016/j.pcad.2014.07.007 25443823

[4] Yusuf S, Hawken S, Ounpuu S, Dans T, Avezum A, Lanas F, et al Effect of potentially modifiable risk factors associated with myocardial infarction in 52 countries (the INTERHEART study): case-control study. Lancet 2004; 364(9438): 937–52. 10.1016/S0140-6736(04)17018-9 15364185

[5] Lim SS, Vos T, Flaxman AD, Danaei G, Shibuya K, Adair-Rohani H, et al A comparative risk assessment of burden of disease and injury attributable to 67 risk factors and risk factor clusters in 21 regions, 1990–2010: a systematic analysis for the Global Burden of Disease Study 2010. Lancet 2012; 380(9859): 2224–60. 10.1016/S0140-6736(12)61766-8 23245609 PMC4156511

[6] Ministerio de Salud de Argentina. Estadísticas vitales. Información básica–Año 2010. Serie 5, Nro. 54. Dirección de Estadísticas e Información en Salud. Buenos Aires. República Argentina. 2011.

[7] Rubinstein A, Colantonio L, Bardach A, Caporale J, García Martí S, Kopitowski K, et al Estimate of the cardiovascular disease burden attributable to modifiable risk factors in Argentina. Rev Panam Salud Publica 2010 27(4): 237–45. 20512225 10.1590/s1020-49892010000400001

[8] Cremonte M, Biscarra MA, Conde K, Cherpitel CJ. Epidemiology of alcohol consumption and related problems in Latin American countries: Contributions of psychology. Int J Psychol 2016. 10.1002/ijop.12373 PMC5337453 27594582

[9] Shield KD, Rylett M, Gmel G, Gmel G, Kehoe-Chan TA, Rehm J. Global alcohol exposure estimates by country, territory and region for 2005—a contribution to the Comparative Risk Assessment for the 2010 Global Burden of Disease Study. Addiction (Abingdon, England) 2013; 108(5): 912–22. 10.1111/add.12112 23347092

[10] Insitute of Health Metrics. GBD Heaetmap. Available at http://vizhub.healthdata.org/gbd-compare/. Accessed 15/07/2015. 2015.

[11] Rehm J, Mathers C, Popova S, Thavorncharoensap M, Teerawattananon Y, Patra J. Global burden of disease and injury and economic cost attributable to alcohol use and alcohol-use disorders. The Lancet 2009; 373(9682): 2223–33. 10.1016/S0140-6736(09)60746-7 19560604

[12] Institute for Health Metrics and Evaluation (IHME). (2013). GBD Heatmap. Seattle, WA: IHME, University of Washington Retrieved from http://vizhub.healthdata.org/irank/heat.php. Accessed Dec 10, 2016.

[13] Monteiro M. Alcohol and Public Health in Latin America: how to prevent a health disaster? Adicciones 2013; 25(2): 99–105. 23748937

[14] Eidelman RS, Vignola P, Hennekens CH. Alcohol consumption and coronary heart disease: a causal and protective factor. Seminars in vascular medicine 2002; 2(3): 253–6. 10.1055/s-2002-35393 16222618

[15] Roerecke M, Rehm J. Irregular heavy drinking occasions and risk of ischemic heart disease: a systematic review and meta-analysis. American journal of epidemiology 2010; 171(6): 633–44. 10.1093/aje/kwp451 20142394

[16] Rehm J, Roerecke M. Alcohol, the heart and the cardiovascular system: what do we know and where should we go? Drug and alcohol review 2011; 30(4): 335–7. 10.1111/j.1465-3362.2011.00338.x 21726306

[17] Instituto Nacional de Estadísticas y Censos (INDEC) A. Encuesta Nacional sobre Prevalencias de Consumo de Sustancias Psicoactivas (ENPreCoSP). 2011. http://www.indec.gob.ar/nivel4_default.asp?id_tema_1=4&id_tema_2=32&id_tema_3=67 (accessed 30th April 2016).

[18] Single E, Robson L, Rehm J, Xie X. Morbidity and mortality attributable to alcohol, tobacco, and illicit drug use in Canada. American journal of public health 1999; 89(3): 385–90. 10076491 10.2105/ajph.89.3.385 PMC1508614

[19] Gutjahr E, Gmel G, Rehm J. Relation between average alcohol consumption and disease: an overview. European addiction research 2001; 7(3): 117–27. 11509842 10.1159/000050729

[20] Ridolfo B, Stevenson C (2001) The quantification of drug-caused mortality and morbidity in Australia 1998. Australian Institute of Health and Welfare, Canberra Available at: [http://www.aihw.gov.au/WorkArea/DownloadAsset.aspx?id=6442459313] accessed: Jan 2015.

[21] Corrao G, Bagnardi V, Zambon A, La Vecchia C. A meta-analysis of alcohol consumption and the risk of 15 diseases. Preventive medicine 2004; 38(5): 613–9. 10.1016/j.ypmed.2003.11.027 15066364

[22] Rehm J, Room R, Monteiro M, Gmel G, Graham K, al e. Alcohol use In: Ezzati M, Lopez AD, Rogers A, Murray CJL, eds. Comparative quantification of health risks: Global and regional burden of disease attributable to selected major risk factors. Geneva: WHO pp 959–1108. 2004.

[23] Bagnardi V, Scotti L, La Vecchia C, W Z. Binge drinking and cardiovascular disease: a meta-analysis. J Epidemiol Community Med 2008; 63: 615–9. 10.1136/jech.2007.065607 18559444

[24] O'Donnell MJ, Xavier D, Liu L, Zhang H, Chin SL, Rao-Melacini P, et al Risk factors for ischaemic and intracerebral haemorrhagic stroke in 22 countries (the INTERSTROKE study): a case-control study. Lancet 2010; 376(9735): 112–23. 10.1016/S0140-6736(10)60834-3 20561675

[25] Mazzaglia G, Britton AR, Altmann DR, Chenet L. Exploring the relationship between alcohol consumption and non-fatal or fatal stroke: a systematic review. Addiction (Abingdon, England) 2001; 96(12): 1743–56. 10.1046/j.1360-0443.2001.961217434.x 11784467

[26] Argentinean Ministry of Health—Directorate of Statistics and Health Information (DEIS). Argentina 2010 vital statistics. Available: http://www.deis.gov.ar/Publicaciones/Archivos/Serie5Nro54.pdf. Accessed november 2014. 2011.

[27] Saposnik G, Del Brutto OH, Iberoamerican Society of Cerebrovascular D. Stroke in South America: a systematic review of incidence, prevalence, and stroke subtypes. Stroke; a journal of cerebral circulation 2003; 34(9): 2103–7. 10.1161/01.STR.0000088063.74250.DB 12907823

[28] Saposnik G, Caplan LR, Gonzalez LA, Baird A, Dashe J, Luraschi A, et al Differences in Stroke Subtypes Among Natives and Caucasians in Boston and Buenos Aires. Stroke; a journal of cerebral circulation 2000; 31(10): 2385–9. 10.1161/01.str.31.10.2385 11022068

[29] Forouzanfar MH, Moran AE, Flaxman AD, Roth G, Mensah GA, Ezzati M, et al Assessing the global burden of ischemic heart disease, part 2: analytic methods and estimates of the global epidemiology of ischemic heart disease in 2010. Global heart 2012; 7(4): 331–42. 10.1016/j.gheart.2012.10.003 23505617 PMC3595103

[30] Ferlay J, Soerjomataram I, Ervik M, Dikshit R, Eser S, Mathers C, et al. GLOBOCAN 2012 v1.0, Cancer Incidence and Mortality Worldwide: IARC CancerBase No. 11 [Internet]. Lyon, France: International Agency for Research on Cancer; 2013. Available from: http://globocan.iarc.fr/, Accessed on 20th August, 2015. 2012.

[31] Barendregt JJ, Van Oortmarssen GJ, Vos T, Murray CJ. A generic model for the assessment of disease epidemiology: the computational basis of DisMod II. Population health metrics 2003; 1(1): 4 10.1186/1478-7954-1-4 12773212 PMC156029

[32] Bazzino O, Diaz R, Tajer C, Paviotti C, Mele E, Trivi M, et al Clinical predictors of in-hospital prognosis in unstable angina: ECLA 3. The ECLA Collaborative Group. Am Heart J 1999; 137(2): 322–31. 9924167 10.1053/hj.1999.v137.93029

[33] Ministry of Health—Directorate of Statistics and Health Information (DEIS). Hospital Discharges Database Argentina 2012.

[34] Murray CJ, Ezzati M, Lopez AD, Rodgers A, Vander Hoorn S. Comparative quantification of health risks conceptual framework and methodological issues. Population health metrics 2003; 1(1): 1 10.1186/1478-7954-1-1 12780936 PMC156894

[35] Murray CJ, Acharya AK. Understanding DALYs (disability-adjusted life years). Journal of health economics 1997; 16(6): 703–30. 10176780 10.1016/s0167-6296(97)00004-0

[36] Salomon JA, Vos T, Hogan DR, Gagnon M, Naghavi M, Mokdad A, et al Common values in assessing health outcomes from disease and injury: disability weights measurement study for the Global Burden of Disease Study 2010. Lancet 2012; 380(9859): 2129–43. 10.1016/S0140-6736(12)61680-8 23245605 PMC10782811

[37] Fox-Rushby JA, Hanson K. Calculating and presenting disability adjusted life years (DALYs) in cost-effectiveness analysis. Health policy and planning 2001; 16(3): 326–31. 11527874 10.1093/heapol/16.3.326

[38] Ministerio de Salud de Argentina. Dirección de Estadística e Información en Salud (DEIS). Base de datos de mortalidad 2010 (microanálisis). Buenos Aires; 2010. 2011.

[39] World Health Organization. Health statistics and information systems. http://www.who.int/healthinfo/global_burden_disease/tools_software/en/. Accessed June 25th, 2015.

[40] Ministerio de Salud de Argentina. Dirección de Estadística e Información en Salud (DEIS). Base de Egresos Hospitalarios 2012 (microanálisis). Buenos Aires; 2014. 2012.

[41] Borras X, Garcia-Moll X, Gomez-Doblas JJ, Zapata A, Artigas R, researchers As. Stable angina in Spain and its impact on quality of life. The AVANCE registry. Revista espanola de cardiologia 2012; 65(8): 734–41. 10.1016/j.recesp.2012.03.011 22739550

[42] Alemán A, Etchepareborda I, Sottano E, Colla C, García I, Abrahín J, et al Efectividad y seguridad de anticoagulación oral y antiagregación luego de un evento cerebrovascular isquémico asociado a fibrilación auricular no valvular. Neurología Argentina 2014; 7(1): 3–10.

[43] Maredza M, Bertram MY, Tollman SM. Disease burden of stroke in rural South Africa: an estimate of incidence, mortality and disability adjusted life years. BMC Neurol 2015; 15: 54 10.1186/s12883-015-0311-7 25880843 PMC4396076

[44] Martin J, Barry J, Goggin D, Morgan K, Ward M, O'Suilleabhain T. Alcohol-attributable mortality in Ireland. Alcohol and alcoholism (Oxford, Oxfordshire) 2010; 45(4): 379–86. 10.1093/alcalc/agq032 20530495

[45] Wong MD, Chung AK, Boscardin WJ, Li M, Hsieh HJ, Ettner SL, et al The contribution of specific causes of death to sex differences in mortality. Public Health Rep 2006; 121(6): 746–54. 10.1177/003335490612100615 17278410 PMC1781916

[46] Connor J, Broad J, Rehm J, Vander Hoorn S, Jackson R. The burden of death, disease, and disability due to alcohol in New Zealand. N Z Med J 2005; 118(1213): U1412 15843841

[47] Borruel M. Estudio de carga de enfermedad: Argentina. - 1a ed.—Buenos Aires: Ministerio de Salud de la Nación, 2010 Available at: http://www.ms.gba.gov.ar/sitios/remediarmasredes/files/2012/09/Estudio-de-carga-de-enfermedad-FESP-Argentina-2010.pdf Last accessed 5 January 2016. 2010.

[48] Feigin VL, Lawes CM, Bennett DA, Barker-Collo SL, Parag V. Worldwide stroke incidence and early case fatality reported in 56 population-based studies: a systematic review. The Lancet Neurology 2009; 8(4): 355–69. 10.1016/S1474-4422(09)70025-0 19233729

[49] Velásqeuz Valdivia A, Cachay C, Munayco C, Poquioma E, Espinoza R, Seclén Y. La carga de enfermedad y lesiones en el Perú. Disponible en http://www.ins.gob.pe/repositorioaps/0/0/jer/ult_inv_evi_cie2010/LacargadelaEmfermedad.pdf Acceso enero 2016. 2008.

[50] Américas Udl. Proyección del Estudio de Carga de Enfermedad en Chile. Primera etapa Insumos para la Construcción de Perfil de Egreso. Available at [http://www.udla.cl/portales/tp9e00af339c16/uploadImg/File/Universidad/UDLA-Proyeccion-Carga-Enfermedad.pdf] accessed January 3rd, 2016. 2008.

[51] Roerecke M, Rehm J. Ischemic heart disease mortality and morbidity rates in former drinkers: a meta-analysis. American journal of epidemiology 2011; 173(3): 245–58. 10.1093/aje/kwq364 21156750 PMC3105267

[52] Chisholm D, Rehm J, Van Ommeren M, Monteiro M. Reducing the global burden of hazardous alcohol use: a comparative cost-effectiveness analysis. J Stud Alcohol. 65:782−93. 2004 15700517 10.15288/jsa.2004.65.782

[53] Anderson P, Chisholm D, Fuhr DC. Effectiveness and cost-effectiveness of policies and programmes to reduce the harm caused by alcohol. Lancet 2009; 373(9682): 2234–46. 10.1016/S0140-6736(09)60744-3 19560605

[54] World Health Organization. The global status report on alcohol and health 2011. Geneva (http://www.who.int/substance_abuse/publications/global_alcohol_report/en/, accessed 14 April 2014).

[55] Robaina K, Babor TF. Alcohol industry marketing strategies in Latin America and the Caribbean: the way forward for policy research. Addiction (Abingdon, England) 2017; 112 Suppl 1: 122–4. 10.1111/add.13625 28070936

[56] Ministry of Health Argentina. Tercera Encuesta Nacional de Factores de Riesgo. Buenos Aires: Ministerio de Salud; 2013 Available at: http://www.msal.gob.ar/images/stories/publicaciones/pdf/11.09.2014-tercer-encuentro-nacional-factores-riesgo.pdf. Accesed December 2nd, 2015. 2014.

