# Supplementary material for: Impact of level and patterns of alcohol drinking on coronary heart disease and stroke burden in Argentina
Source: PLoS One. 2017 Mar 10;12(3):e0173704. doi: 10.1371/journal.pone.0173704 (PMC5345854; doi:10.1371/journal.pone.0173704)
Supplement: S1 Table — a. Distribution of cause-specific deaths attributable to alcohol by age group and by sex. b. Years of life lost (YLL) and years of life with disability (YLD) by age group, gender and health condition. c. Incident CVD cases estimated (fatal and non-fatal). d. Case-fatality rates used for estimating CHD and stroke incident cases. e. Stroke disability distribution from an Argentinean source (Aleman et al, 2014). f. Stroke disability categories and corresponding DWs. g. Number of alcohol-attributable deaths for ischemic heart disease, ischemic stroke and hemorrhagic stroke, by educaction level and gender. Argentina, 2010. (DOCX) [file pone.0173704.s001.docx]

Supplemental Material

Abbreviations:

ACS: Acute Coronary Syndrome

AMI: Acute myocardial infarction

CHD: Coronary Heart Disease

DW: Disability Weighs

S1 Table a. Distribution of cause-specific deaths attributable to alcohol by age group and by sex.

|  | CHD | Ischemic stroke | Hemorrhagic stroke |
| --- | --- | --- | --- |
| 35 to 44 | -91.9 | 0.0 | 85.0 |
| 45 to 59 | -604.7 | 0.6 | 312.1 |
| 60 to 69 | -394.1 | 17.3 | 208.6 |
| 70 to 79 | -220.3 | 43.8 | 149.8 |
| 80+ | -112.5 | 59.4 | 58.3 |
| Men | -1115.4 | 125.5 | 704.8 |
| Women | -308.2 | -4.6 | 109.0 |

S1 Table b. Years of life lost (YLL) and years of life with disability (YLD) by age group, gender and health condition

|  | CHD (AMI & Acute Coronary Syndrome) | | | | | | |
| --- | --- | --- | --- | --- | --- | --- | --- |
|  | YLL (0;0) | | YLD (0;0) | | |  | |
| Age group | Men | Women | | Men | Women | |  |
| 35 to 44 | -3,748 | -1,175 | | -806 | -844 | |  |
| 45 to 59 | -26,498 | -6,309 | | -3,442 | -3,339 | |  |
| 60 to 69 | -16,608 | -3,787 | | -1,256 | -1,072 | |  |
| 70 to 79 | -7,908 | -2,995 | | -328 | -243 | |  |
| 80+ | -2,957 | -2,330 | | -49 | -29 | |  |
| **Total** | -57,719 | -16,596 | | -5,880 | -5,527 | |  |

|  | Stroke (both ischemic and hemorrhagic) | | | | | | |
| --- | --- | --- | --- | --- | --- | --- | --- |
|  | YLL (0;0)* | | YLD (0;0)* | | |  |  |
| Age group | Men | Women | | Men | Women | |  |
| 35 to 44 | 3,092 | 824 | | 4,014 | 1,017 | |  |
| 45 to 59 | 9,458 | 1,900 | | 10,994 | 2,460 | |  |
| 60 to 69 | 4,456 | 452 | | 5,724 | 588 | |  |
| 70 to 79 | 2,454 | 238 | | 2,814 | 291 | |  |
| 80+ | 656 | 75 | | 600 | 65 | |  |
| **Total** | 20,116 | 3,488 | | 24,146 | 4,421 | |  |

Discount (r) = 0% and Social ponderation (K) = 0

S1 Table c. Incident CVD cases estimated (fatal and non-fatal)

| Total Population  (2010 Census) | | ACS | | AMI | | Total Stroke | |  |
| --- | --- | --- | --- | --- | --- | --- | --- | --- |
|  | Male | Female | Male | Female | Male | Female | Male | Female |
| 35 | 282,604 | 293,989 | 17 | 36 | 23 | 30 | 69 | 45 |
| 36 | 262,693 | 274,036 | 25 | 50 | 31 | 24 | 112 | 65 |
| 37 | 259,598 | 269,801 | 17 | 53 | 39 | 24 | 80 | 65 |
| 38 | 256,537 | 267,319 | 17 | 48 | 42 | 47 | 112 | 65 |
| 39 | 250,096 | 261,762 | 40 | 17 | 73 | 18 | 136 | 65 |
| 40 | 248,912 | 260,630 | 33 | 35 | 70 | 29 | 176 | 120 |
| 41 | 228,038 | 238,820 | 50 | 69 | 89 | 57 | 125 | 143 |
| 42 | 222,817 | 234,737 | 58 | 41 | 78 | 45 | 151 | 126 |
| 43 | 213,101 | 225,598 | 50 | 34 | 88 | 73 | 193 | 132 |
| 44 | 213,019 | 225,103 | 66 | 17 | 113 | 111 | 198 | 181 |
| 45 | 220,704 | 232,876 | 74 | 16 | 134 | 66 | 239 | 252 |
| 46 | 215,428 | 227,881 | 25 | 156 | 117 | 102 | 286 | 166 |
| 47 | 215,468 | 228,471 | 82 | 16 | 177 | 43 | 297 | 160 |
| 48 | 211,989 | 222,824 | 114 | 63 | 149 | 100 | 312 | 269 |
| 49 | 203,879 | 216,830 | 121 | 140 | 216 | 140 | 333 | 259 |
| 50 | 212,341 | 226,436 | 145 | 122 | 172 | 138 | 469 | 341 |
| 51 | 194,858 | 209,182 | 184 | 60 | 240 | 121 | 572 | 336 |
| 52 | 194,065 | 207,388 | 144 | 104 | 276 | 153 | 506 | 377 |
| 53 | 194,635 | 207,971 | 182 | 160 | 365 | 199 | 474 | 383 |
| 54 | 190,297 | 205,820 | 174 | 115 | 360 | 196 | 517 | 365 |
| 55 | 187,643 | 204,200 | 251 | 70 | 403 | 258 | 555 | 441 |
| 56 | 181,432 | 197,213 | 343 | 111 | 379 | 194 | 737 | 430 |
| 57 | 178,927 | 194,843 | 271 | 190 | 429 | 204 | 686 | 430 |
| 58 | 175,414 | 191,968 | 346 | 160 | 457 | 284 | 848 | 385 |
| 59 | 170,154 | 187,156 | 382 | 170 | 484 | 213 | 742 | 480 |
| 60 | 173,319 | 194,935 | 327 | 268 | 473 | 226 | 876 | 424 |
| 61 | 154,790 | 171,821 | 369 | 162 | 462 | 263 | 1,092 | 553 |
| 62 | 151,211 | 171,678 | 352 | 208 | 481 | 306 | 1,074 | 512 |
| 63 | 144,736 | 164,058 | 408 | 119 | 532 | 267 | 1,177 | 553 |
| 64 | 136,858 | 157,784 | 538 | 222 | 513 | 253 | 1,098 | 616 |
| 65 | 138,149 | 162,109 | 431 | 182 | 594 | 258 | 1,092 | 806 |
| 66 | 122,707 | 145,289 | 456 | 244 | 545 | 311 | 1,132 | 624 |
| 67 | 115,871 | 139,122 | 509 | 249 | 545 | 296 | 1,099 | 666 |
| 68 | 109,009 | 132,571 | 412 | 253 | 545 | 267 | 1,187 | 801 |
| 69 | 102,833 | 125,401 | 542 | 246 | 549 | 336 | 1,209 | 760 |
| 70 | 105,949 | 134,546 | 459 | 280 | 578 | 380 | 1,380 | 921 |
| 71 | 91,197 | 116,686 | 468 | 243 | 560 | 369 | 1,311 | 1,019 |
| 72 | 84,638 | 111,644 | 429 | 349 | 536 | 437 | 1,382 | 1,218 |
| 73 | 80,445 | 108,495 | 485 | 375 | 534 | 406 | 1,412 | 1,129 |
| 74 | 76,209 | 106,088 | 619 | 329 | 464 | 338 | 1,412 | 1,284 |
| 75 | 75,096 | 106,217 | 500 | 345 | 585 | 416 | 1,503 | 1,207 |
| 76 | 65,769 | 95,524 | 526 | 334 | 420 | 417 | 1,423 | 1,413 |
| 77 | 62,633 | 94,006 | 545 | 364 | 423 | 423 | 1,441 | 1,443 |
| 78 | 61,294 | 94,629 | 556 | 414 | 535 | 474 | 1,472 | 1,595 |
| 79 | 56,689 | 89,802 | 480 | 378 | 459 | 517 | 1,360 | 1,766 |
| 80 | 52,487 | 90,998 | 485 | 497 | 520 | 584 | 1,486 | 1,560 |
| 81 | 44,177 | 76,866 | 605 | 473 | 436 | 567 | 1,271 | 1,512 |
| 82 | 39,776 | 71,857 | 447 | 477 | 413 | 493 | 1,076 | 1,790 |
| 83 | 34,268 | 65,651 | 428 | 401 | 395 | 504 | 1,022 | 1,829 |
| 84 | 30,036 | 59,800 | 375 | 532 | 341 | 538 | 1,134 | 1,638 |
| 85 | 26,288 | 54,171 | 380 | 531 | 373 | 504 | 988 | 1,825 |
| 86 | 22,142 | 47,092 | 268 | 488 | 294 | 523 | 788 | 1,288 |
| 87 | 18,264 | 40,858 | 307 | 441 | 330 | 473 | 582 | 1,323 |
| 88 | 14,594 | 34,772 | 307 | 457 | 271 | 426 | 610 | 1,166 |
| 89 | 11,560 | 28,596 | 295 | 392 | 218 | 370 | 483 | 1,082 |
| 90 | 9,348 | 25,159 | 155 | 337 | 152 | 312 | 373 | 839 |

S1 Table d. Case-fatality rates used for estimating CHD and stroke incident cases

| AMI^#^ | | ACS | | Stroke | |  |
| --- | --- | --- | --- | --- | --- | --- |
|  | Male | Female | Male | Female | Male | Female |
| 35 | 35.4% | 16.5% | 11.8% | 5.5% | 23.1% | 22.4% |
| 36 | 35.4% | 16.7% | 11.8% | 5.6% | 12.5% | 18.4% |
| 37 | 35.5% | 16.8% | 11.8% | 5.6% | 12.5% | 18.4% |
| 38 | 35.6% | 17.0% | 11.9% | 5.7% | 12.5% | 18.4% |
| 39 | 35.7% | 17.1% | 11.9% | 5.7% | 12.5% | 18.4% |
| 40 | 35.9% | 17.3% | 11.9% | 5.8% | 12.5% | 18.4% |
| 41 | 36.0% | 17.5% | 12.0% | 5.8% | 19.2% | 18.2% |
| 42 | 36.1% | 17.7% | 12.0% | 5.9% | 19.2% | 18.2% |
| 43 | 36.2% | 17.9% | 12.1% | 6.0% | 19.2% | 18.2% |
| 44 | 36.3% | 18.1% | 12.1% | 6.0% | 19.2% | 18.2% |
| 45 | 36.5% | 18.3% | 12.2% | 6.1% | 19.2% | 18.2% |
| 46 | 36.6% | 18.6% | 12.2% | 6.2% | 19.2% | 19.3% |
| 47 | 36.8% | 18.8% | 12.3% | 6.3% | 19.2% | 19.3% |
| 48 | 36.9% | 19.1% | 12.3% | 6.4% | 19.2% | 19.3% |
| 49 | 37.1% | 19.3% | 12.4% | 6.4% | 19.2% | 19.3% |
| 50 | 37.3% | 19.6% | 12.4% | 6.5% | 19.2% | 19.3% |
| 51 | 37.4% | 19.9% | 12.5% | 6.6% | 18.4% | 17.2% |
| 52 | 37.6% | 20.2% | 12.5% | 6.7% | 18.4% | 17.2% |
| 53 | 37.8% | 20.6% | 12.6% | 6.9% | 18.4% | 17.2% |
| 54 | 38.0% | 20.9% | 12.7% | 7.0% | 18.4% | 17.2% |
| 55 | 38.3% | 21.3% | 12.7% | 7.1% | 18.4% | 17.2% |
| 56 | 38.5% | 21.7% | 12.8% | 7.2% | 17.9% | 17.9% |
| 57 | 38.7% | 22.1% | 12.9% | 7.4% | 17.9% | 17.9% |
| 58 | 39.0% | 22.5% | 13.0% | 7.5% | 17.9% | 17.9% |
| 59 | 39.2% | 23.0% | 13.1% | 7.7% | 17.9% | 17.9% |
| 60 | 39.5% | 23.5% | 13.2% | 7.8% | 17.9% | 17.9% |
| 61 | 39.8% | 24.0% | 13.3% | 8.0% | 16.6% | 17.4% |
| 62 | 40.1% | 24.5% | 13.4% | 8.2% | 16.6% | 17.4% |
| 63 | 40.4% | 25.1% | 13.5% | 8.4% | 16.6% | 17.4% |
| 64 | 40.7% | 25.7% | 13.6% | 8.6% | 16.6% | 17.4% |
| 65 | 41.1% | 26.3% | 13.7% | 8.8% | 16.6% | 17.4% |
| 66 | 41.5% | 27.0% | 13.8% | 9.0% | 18.1% | 19.2% |
| 67 | 41.8% | 27.7% | 13.9% | 9.2% | 18.1% | 19.2% |
| 68 | 42.2% | 28.4% | 14.1% | 9.5% | 18.1% | 19.2% |
| 69 | 42.7% | 29.2% | 14.2% | 9.7% | 18.1% | 19.2% |
| 70 | 43.1% | 30.0% | 14.4% | 10.0% | 18.1% | 19.2% |
| 71 | 43.6% | 30.9% | 14.5% | 10.3% | 19.8% | 18.2% |
| 72 | 44.0% | 31.8% | 14.7% | 10.6% | 19.8% | 18.2% |
| 73 | 44.5% | 32.8% | 14.8% | 10.9% | 19.8% | 18.2% |
| 74 | 45.1% | 33.8% | 15.0% | 11.3% | 19.8% | 18.2% |
| 75 | 45.6% | 34.8% | 15.2% | 11.6% | 19.8% | 18.2% |
| 76 | 46.2% | 36.0% | 15.4% | 12.0% | 22.1% | 20.4% |
| 77 | 46.8% | 37.1% | 15.6% | 12.4% | 22.1% | 20.4% |
| 78 | 47.5% | 38.4% | 15.8% | 12.8% | 22.1% | 20.4% |
| 79 | 48.1% | 39.7% | 16.0% | 13.2% | 22.1% | 20.4% |
| 80 | 48.8% | 41.1% | 16.3% | 13.7% | 22.1% | 20.4% |
| 81 | 49.6% | 42.5% | 16.5% | 14.2% | 24.1% | 23.0% |
| 82 | 50.3% | 44.1% | 16.8% | 14.7% | 24.1% | 23.0% |
| 83 | 51.1% | 45.7% | 17.0% | 15.2% | 24.1% | 23.0% |
| 84 | 52.0% | 47.4% | 17.3% | 15.8% | 24.1% | 23.0% |
| 85 | 52.9% | 49.2% | 17.6% | 16.4% | 24.1% | 23.0% |
| 86 | 53.8% | 51.1% | 17.9% | 17.0% | 29.2% | 28.7% |
| 87 | 54.8% | 53.1% | 18.3% | 17.7% | 29.2% | 28.7% |
| 88 | 55.8% | 55.2% | 18.6% | 18.4% | 29.2% | 28.7% |
| 89 | 56.9% | 57.4% | 19.0% | 19.1% | 29.2% | 28.7% |
| 90 | 58.0% | 59.7% | 19.3% | 19.9% | 29.2% | 28.7% |
| 91 | 59.1% | 62.1% | 19.7% | 20.7% | 40.0% | 35.0% |
| 92 | 60.4% | 64.7% | 20.1% | 21.6% | 40.0% | 35.0% |
| 93 | 61.7% | 67.5% | 20.6% | 22.5% | 40.0% | 35.0% |
| 94 | 63.0% | 70.3% | 21.0% | 23.4% | 40.0% | 35.0% |
| 95 | 64.4% | 73.4% | 21.5% | 24.5% | 40.0% | 35.0% |
| 96 | 65.9% | 76.5% | 22.0% | 25.5% | 40.0% | 35.0% |
| 97 | 67.5% | 79.9% | 22.5% | 26.6% | 40.0% | 35.0% |
| 98 | 69.1% | 83.4% | 23.0% | 27.8% | 40.0% | 35.0% |
| 99 | 70.8% | 87.2% | 23.6% | 29.1% | 40.0% | 35.0% |

^#^ For AMI, the overall estimate is that of GBD 2010 for Southern Latin America, for men the case fatality rate is 0.44 and 0.38 for women. In order to project age-specific rates, we use the age distribution national hospital discharges 2012, and the corresponding official AMI deaths. Then we adjusted the resulting rates by age proportionately, in order to obtain the same number of events predicted by global GBD rates. We assumed that the age distribution of non-fatal events of this database is representative of Argentina.

S1 Table e. Stroke disability distribution from an Argentinean source (Aleman et al, 2014)

| Modified Ranking Scale at discharge (mRS) | Patients (N)  (Alemán et al, 2014) | Percentage (%) |
| --- | --- | --- |
| 0 | 15 | 13,2 |
| 1 | 20 | 17,5 |
| 2 | 24 | 21,1 |
| 3 | 21 | 18,4 |
| 4 | 15 | 13,2 |
| 5 | 19 | 16,7 |
| 6 | 0 | 0,0 |
| Total | 114 | 100 |
|  |  |  |

Alemán A, Etchepareborda I, Sottano E, Colla C, García I, Abrahín J et al. Efectividad y seguridad de anticoagulación oral y antiagregación luego de un evento cerebrovascular isquémico asociado a fibrilación auricular no valvular. Neurología Argentina. 2014;7(1):3-10.

S1 Table f. Stroke disability categories and corresponding DWs

| Category | Salomon 2012 |
| --- | --- |
|  |  |
| Mild | 0,0 |
| Moderate | 0,1 |
| Moderate plus cognitive impairment | 0,3 |
| Severe | 0,5 |
| Severe plus cognitive impairment | 0,6 |
| Weighted DW | **0,3** |
|  |  |

We multiplied the DW of each category of severity by the corresponding distribution of cases reported in Argentinean shown in Table e. We mapped each GDB DWs (Salomon 2012) to their corresponding category of the modified Rankin Scale (mRS) assuming similar distribution of severity on the basis of the GBD sequel definition. The mRS is a commonly used scale for measuring the degree of disability or dependence in the daily activities of people who have suffered a stroke or other causes of neurological disability. The slight difference between the definition of disability on mRS and categories in GBD 2010 was regarded as acceptable by a consensus of members of an international collaborative stroke expert group from a study performed in South Africa, that used the same methodology.^36^

Salomon JA, Vos T, Hogan DR, Gagnon M, Naghavi M, Mokdad A et al. Common values in assessing health outcomes from disease and injury: disability weights measurement study for the Global Burden of Disease Study 2010. Lancet. 2012;380(9859):2129-43. doi:10.1016/S0140-6736(12)61680-8.

CHD disability weights estimation

All disability weights (DW) were obtained from Salomon et al, 2012. We calculated a unique weight for each of the conditions included (AMI, ACS). For AMI we weighted the DW for days 1-2 and days 3-28 according to the distribution of length of stay of hospitalizations due to AMI in the public sector. For ACS we estimated DWs from a large Spanish coronary heart disease registry (AVANCE Registry, Borras et al, 2012)

Resulting DWs

1. Acute myocardial infarction: days 1-2 = 0.422 (IC95% 0.284-0.566)

2. Acute myocardial infarction: days 3-28 = 0.056 (0.035-0.082)

**Weighted DW for AMI 0,169158648**

DWs according to the AVANCE Registry (Spain)

DW for ACS Patient Distribution

0,037 mild 35%

0,066 moderate 48%

0,167 severe 18%

**Weighted DW for ACS: 0,073525**

Borras X, Garcia-Moll X, Gomez-Doblas JJ, Zapata A, Artigas R, researchers As. Stable angina in Spain and its impact on quality of life. The AVANCE registry. Revista espanola de cardiologia. 2012;65(8):734-41. doi:10.1016/j.recesp.2012.03.011.

S1 Table g. Number of alcohol-attributable deaths for ischemic heart disease, ischemic stroke and hemorrhagic stroke, by educaction level and gender. Argentina, 2010.

|  |  | Men | Women | Both genders |
| --- | --- | --- | --- | --- |
|  | Education level | Total | Total | Total |
|  |  | Attributable | Attributable | Attributable |
|  |  | Deaths (n) | Deaths (n) | Deaths (n) |
| Coronary Heart Disease | Elementary school incomplete or lower | -87.2 | -20.4 | -102.7 |
|  | Complete Elementary School and Incomplete High School | -468.2 | -113.9 | -561.4 |
|  | Complete High School | -269.0 | -74.3 | -343.3 |
|  | Tertiary or University incomplete or higher | -283.8 | -100.6 | -414.0 |
|  | Special schooling | -7.1 | 1.0 | -2.0 |
|  | Total | -1115.3 | -308.2 | -1423.5 |
| Ischemic Stroke | Elementary school incomplete or lower | 9.8 | -0.3 | 8.7 |
|  | Complete Elementary School and Incomplete High School | 52.8 | -1.7 | 47.8 |
|  | Complete High School | 30.3 | -1.1 | 29.2 |
|  | Tertiary or University incomplete or higher | 32.0 | -1.5 | 35.2 |
|  | Special schooling | 0.8 | 0.0 | 0.2 |
|  | Total | 125.7 | -4.5 | 121.2 |
| Hemorrhagic Stroke | Elementary school incomplete or lower | 55.1 | 7.2 | 58.7 |
|  | Complete Elementary School and Incomplete High School | 295.9 | 40.3 | 321.0 |
|  | Complete High School | 170.0 | 26.3 | 196.3 |
|  | Tertiary or University incomplete or higher | 179.4 | 35.6 | 236.7 |
|  | Special schooling | 4.5 | -0.4 | 1.2 |
|  | Total | 704.9 | 109 | 813.9 |
|  |  |  |  |  |

**Supporting Information Captions**

**S1 Table a. Distribution of cause-specific deaths attributable to alcohol by age group and by sex.**

**S1 Table b. Years of life lost (YLL) and years of life with disability (YLD) by age group, gender and health condition**

**S1 Table c. Incident CVD cases estimated (fatal and non-fatal)**

**S1 Table d. Case-fatality rates used for estimating CHD and stroke incident cases**

**S1 Table e. Stroke disability distribution from an Argentinean source (Aleman et al, 2014)**

**S1 Table f. Stroke disability categories and corresponding DWs**

**S1 Table g. Number of alcohol-attributable deaths for ischemic heart disease, ischemic stroke and hemorrhagic stroke, by educaction level and gender. Argentina, 2010.**
